# Correction: Synthesis, characterization, DNA interaction, and anticancer studies of novel *facial* tricarbonylrhenium(i) complexes with uracil-derived ligands

**DOI:** 10.1039/d5ra90121a

**Published:** 2025-10-27

**Authors:** Shabaaz Abdullah, Candace Davison, Christie Jane Smit, Phiwokuhle Mbatha, Jo-Anne de la Mare, Irvin Noel Booysen

**Affiliations:** a School of Chemistry and Physics, University of KwaZulu-Natal Pietermaritzburg 3201 South Africa booyseni@ukzn.ac.za; b Department of Biochemistry, Microbiology and Bioinformatics, Faculty of Science, Rhodes University PO Box 94 Grahamstown 6140 South Africa j.delamare@ru.ac.za

## Abstract

Correction for ‘Synthesis, characterization, DNA interaction, and anticancer studies of novel *facial* tricarbonylrhenium(i) complexes with uracil-derived ligands’ by Shabaaz Abdullah *et al.*, *RSC Adv.*, 2025, **15**, 34635–34642, https://doi.org/10.1039/D5RA05387K.

The authors regret that the name of one of the authors (Candace Davison) was shown incorrectly in the author contributions section. In addition, the author contributions of Candace Davison and Christie Jane Smit were incorrectly given. Finally, Andile Kathi was incorrectly included in the author contributions section, as this individual was not an author of the manuscript.

The corrected author contributions are as follows:

Shabaaz Abdullah (SA): writing – original draft preparation, methodology, formal analysis, investigation, data curation, visualization. Candace Davison (CD): writing – original draft preparation, methodology, formal analysis, investigation, data curation, visualization. Christie Jane Smit (CJS): methodology, investigation. Phiwokuhle Mbatha (PM): formal analysis, investigation, data curation, visualization. Jo-Anne de la Mare (JDLM): funding acquisition, conceptualization, writing – review & editing, co-funding and resources, supervision of CJS, host of CA. Irvin Noel Booysen (INB): funding acquisition, conceptualization, writing – review & editing, methodology, co-funding and resources, supervision of SA and PM.

The Royal Society of Chemistry apologises for these errors and any consequent inconvenience to authors and readers.

